# Clinical Perfectionism in Anorexia Nervosa: Associations with Eating Disorders’ Specific Symptomatology and General Psychological Symptoms in Italian Young Adult Women

**DOI:** 10.3390/jcm15103823

**Published:** 2026-05-15

**Authors:** Anna Guerrini Usubini, Ilaria Bastoni, Maria Gobetti, Adele Bondesan, Diana Caroli, Leonardo Mendolicchio, Gianluca Castelnuovo, Alessandro Sartorio

**Affiliations:** 1 Experimental Laboratory for Auxo-Endocrinological Research, Istituto Auxologico Italiano, Istituto di Ricovero e Cura a Carattere Scientifico (IRCCS), 28824 Piancavallo-Verbania, Italysartorio@auxologico.it (A.S.); 2 Psychology Research Laboratory, Istituto Auxologico Italiano, Istituto di Ricovero e Cura a Carattere Scientifico (IRCCS), 20145 Milan, Italy; 3 Division of Eating and Nutrition Disorders, Istituto Auxologico Italiano, Istituto di Ricovero e Cura a Carattere Scientifico (IRCCS), 28824 Piancavallo-Verbania, Italy

**Keywords:** perfectionism, eating disorders, general psychological symptoms, anorexia nervosa

## Abstract

**Background/Objectives**: Existing literature has consistently identified perfectionism as a core feature of anorexia nervosa (AN). However, much of the research has focused primarily on its association with eating disorder-related symptoms. At the same time, less attention has been paid to understanding how perfectionism affects the overall psychological functioning of individuals with eating disorders, and in particular AN. The present study aimed at investigating the association between perfectionism and both eating disorder-specific symptomatology and general psychological symptoms in a sample of young adult Italian women diagnosed with AN. **Methods**: Thirty-five Italian females diagnosed with AN (mean age ± SD: 21.9 ± 4.18 years; mean body mass index: body mass index ± SD: 14.5 ± 1.77 kg/m^2^) were consecutively recruited at the Division of Eating and Nutrition Disorders, Istituto Auxologico Italiano, IRCCS, Piancavallo-Verbania, a third-level clinical centre for the rehabilitation of obesity and eating disorders located in northern Italy. Once informed about the research, screened for inclusion/exclusion criteria, and enrolled, participants were asked to complete the Eating Disorder Inventory—third edition, the Symptoms Checklist-90 revised, and the Frost Multidimensional Perfectionism Scale to collect demographic and clinical variables of interest for the study. Three hierarchical linear regression models were tested to explore the role of perfectionism in both eating disorder-specific symptomatology and general psychological symptoms. **Results**: The analyses showed a significant influence of perfectionism in all tested models, suggesting that perfectionism was significantly associated with both eating disorder-specific symptomatology and general psychological symptoms in a sample of young adult Italian women diagnosed with AN. **Conclusions**: The findings of this study provide further support for the role of perfectionism in the psychopathology of eating disorders, particularly among young adult women with AN. Consistent with prior research, perfectionism was associated with both disorder-specific symptoms and broader psychological symptoms. Notably, its association remained robust even after controlling for key variables such as age and body mass index, underscoring its potential as an independent risk factor.

## 1. Introduction

Anorexia nervosa (AN) is a severe psychiatric disorder characterized by persistent restriction of energy intake, intense fear of gaining weight, and a disturbance in the perception of body weight or shape [1] that belongs to the spectrum of eating disorders (EDs), which also include, among others, bulimia nervosa (BN) and binge eating disorders (BEDs).

Although each disorder presents with distinct clinical features, a growing body of literature highlights the presence of shared underlying mechanisms across EDs. According to the transdiagnostic theory proposed by Fairburn and colleagues [2], a core psychopathological feature common to these conditions is a dysfunctional system of negative self-evaluation, in which self-worth is predominantly determined by body weight, shape, and their control. This overvaluation contributes to the maintenance of maladaptive eating behaviours, such as extreme dietary restriction in AN, or in cycles of binge eating with or without consequent compensatory behaviours, as in BN and BED. The model further suggests that additional maintenance mechanisms can interact with this dysfunctional self-evaluation system. Among these processes, clinical perfectionism has received empirical attention.

Perfectionism refers to the tendency to base one’s self-worth largely on the attainment of excessively high standards, despite adverse consequences and repeated difficulties in achieving them [3]. Empirical evidence consistently showed that individuals with AN exhibit significantly elevated levels of perfectionism compared to both healthy controls and other psychiatric populations [4,5,6]. In particular, perfectionism can influence eating behaviours through rigid cognitive and behavioural control processes that promote dietary restriction. Individuals with high perfectionistic tendencies often set excessively high standards and evaluate self-worth in terms of self-control and achievement. Within this framework, food intake and body weight may become domains in which perfectionistic standards are applied [7].

Recent research has further highlighted that higher levels of perfectionism in AN are associated with greater symptom severity, specific personality traits, and a more complex clinical presentation, suggesting a role for perfectionism in reinforcing rigid dietary rules and cognitive inflexibility [5]. In addition, perfectionism has been linked to obsessive–compulsive features and dysfunctional beliefs, which may indirectly contribute to disordered eating behaviours and sustain the disorder over time [8].

Beyond its relationship with ED-specific symptoms, clinical perfectionism is increasingly recognized as a transdiagnostic process associated with a variety of mental disorders [9,10]. Meta-analytic evidence demonstrates that perfectionism is consistently related to depression, general anxiety, and obsessive–compulsive tendencies across adult populations [11]. This perspective considers perfectionism not only as a key maintaining factor for ED symptomatology but also as a broader vulnerability trait that may exacerbate overall psychological distress and, in turn, influence ED severity [12].

Perfectionism can be particularly problematic in young adulthood. This is a particular developmental age marked by significant life transitions and psychosocial challenges, including identity consolidation and increasing autonomy [13].

Research has shown that this period of life involves substantial personality development and maturation processes. At the same time, evidence suggests that levels of perfectionism have increased across generations, with contemporary young adults reporting greater self-imposed expectations and stronger drives toward flawlessness compared to previous cohorts [14]. High levels of perfectionistic concerns and standards in young adults have been linked to elevated psychological distress, particularly during critical transitions such as entering university, a context often associated with uncertainty regarding career choices and decision-making difficulties [15].

Although ED typically emerges during adolescence, it was observed that a substantial proportion of young adults aged 18–25 either suffer from an eating disorder or exhibit subclinical symptoms, suggesting that this group may be at heightened risk. This is particularly evident among young women, where objectification and self-objectification are associated with body shame and symptoms of eating disorders [16,17]. In the Italian context, perfectionism has been increasingly recognized as a relevant transdiagnostic factor associated with ED, particularly in relation to family criticism, sociocultural pressures, and the internalization of high standards of achievement and body image ideals [18].

While existing literature has consistently identified perfectionism as a core feature of AN, much of the research has focused primarily on its association with ED-related symptoms. Less attention has been paid to understanding how perfectionism affects the overall psychological functioning of individuals with ED, and in particular, AN.

By adopting a more integrative perspective, simultaneously examining perfectionism in relation to both the severity of eating disorder symptoms and broader psychological maladjustment, the present study aims to investigate the association between perfectionism and both eating disorder-specific symptomatology and general psychological symptoms in a sample of young adult Italian women diagnosed with AN.

## 2. Materials and Methods

### 2.1. Participants and Procedures

The participants were 35 Italian females diagnosed with AN consecutively recruited at the Division of Eating and Nutrition Disorders, Istituto Auxologico Italiano, IRCCS, Piancavallo-Verbania, a third-level clinical centre for the rehabilitation of obesity and ED, in northern Italy.

Inclusion criteria were: (i) female sex; (ii) age between 18 and 35 years; (iii) a diagnosis of AN according to the Diagnostic and Statistical Manual of Mental Disorders, Fifth Edition, assessed using the Structured Clinical Interview–Clinical Version 5 (SCID-5 CV), and (iii) providing written informed consent to participate. Participants were excluded if they had any comorbid psychiatric disorder diagnosed according to the DSM-5 criteria or any other medical condition that could compromise their participation in the study. The Structured Clinical Interview for DSM-5 (SCID-5), used as a screening tool for psychiatric diagnoses, was conducted at admission to the hospital by a clinical psychologist.

Once informed about the research, screened for the inclusion/exclusion criteria, and enrolled, participants were asked to complete self-report questionnaires to collect demographic and clinical variables of interest for the study.

The study was approved by the Ethical Committee of Istituto Auxologico Italiano, IRCCS, Milan, Italy (Ethical Committee code: 2019_10_22_03, date of approval: 22 October 2019). All procedures were conducted in accordance with the Declaration of Helsinki of 1975 and its later amendments or comparable ethical standards.

### 2.2. Measures

Demographic information (age) was self-reported. Anthropometric data were collected by the internal medical staff. Weight and height were measured to calculate body mass index (BMI) using the proper formula (kg/m^2^). Standing height was determined by a Harpenden Stadiometer (Holtain Limited, Crymych, Dyfed, UK). Weight was measured to the nearest 0.1 kg using an electronic scale (RoWU 150, Wunder Sa.bi., Trezzo sull’Adda, Milan, Italy). Clinical data were collected using the following questionnaires.

Eating Disorder Inventory—Third Edition (EDI 3; [19]): It is a widely used self-report questionnaire designed to assess ED-specific symptomatology. The instrument consists of 91 items rated on a 6-point Likert scale composing 12 primary scales that assess eating disorder-specific symptoms and related psychological constructs. In addition, EDI 3 provides several composite scores summarizing broader dimensions of eating disorder risk and associated psychological functioning. For the purposes of the present study, two composite indices were considered: the Eating Disorder Risk Composite (EDRC) and the General Psychological Maladjustment Composite (GPMC). The EDRC reflects core eating disorder symptomatology and includes the drive for thinness, bulimia, and body dissatisfaction subscales. The GPMC assesses broader psychological maladjustment related to eating disorders and comprises nine psychological scales, including low self-esteem, interpersonal insecurity, interpersonal alienation, interoceptive deficits, emotional dysregulation, perfectionism, asceticism, and maturity fears. Higher scores on both composites indicate greater levels of eating disorder-related risk and psychological maladjustment. The reliability coefficients of the scales range from 0.83 to 0.90, and test–retest reliability coefficients for the various composite scales are between 0.84 and 0.87. The Italian version of EDI 3 has demonstrated very good day test–retest reliability, cross-informant agreement, and a good discriminating validity. In our sample, the internal consistency of the EDRC and GPMC was α = 0.850 and α = 0.957, respectively.

Symptoms Checklist-90 Revised (SCL-90-R; [20]), a widely used self-report instrument designed to evaluate general psychological symptoms experienced during the previous week. The questionnaire consists of 90 items rated on a 5-point Likert scale ranging from 0 (“not at all”) to 4 (“extremely”). The SCL-90-R is composed of nine primary symptom dimensions, including somatization, obsessive–compulsive symptoms, interpersonal sensitivity, depression, anxiety, hostility, phobic anxiety, paranoid ideation, and psychoticism, as well as several global indices of psychological distress. For the purposes of the present study, the Global Severity Index (GSI) was used as the main indicator of overall psychological distress. The GSI represents the average score across all 90 items and is considered the most sensitive single indicator of the intensity of global psychological symptomatology. Higher scores reflect greater levels of overall psychological distress. The Italian version of the SCL-90-R has demonstrated satisfactory psychometric properties in large Italian community samples, including good internal consistency, with Cronbach’s alpha coefficients ranging from 0.70 to 0.96. Studies also supported the instrument’s validity as a reliable measure of general psychological distress in both adolescents and adults [21]. In our sample, the internal consistency of the GSI was α = 0.972.

Frost Multidimensional Perfectionism Scale (FMPS; [22,23]) is a widely used self-report questionnaire developed to measure multiple dimensions of perfectionism conceptualized as a multidimensional personality trait. The scale consists of 35 items rated on a 5-point Likert scale ranging from 1 (“strongly disagree”) to 5 (“strongly agree”). The FMPS evaluates six dimensions of perfectionism: concern over mistakes, personal standards, parental expectations, parental criticism, doubts about actions, and organization. Higher scores indicate greater levels of perfectionistic tendencies. For the purposes of the present study, the total FMPS score was used as an overall indicator of perfectionism, with higher scores reflecting greater global levels of perfectionistic traits. The Italian version of FMPS demonstrated satisfactory psychometric properties, including good internal consistency and construct validity. Factor analyses supported a multidimensional structure of perfectionism, with positive and significant correlations between FMPS dimensions and the EDI 2 perfectionism scale, supporting convergent validity in Italian samples. In our sample, the internal consistency of the FPMS was α = 0.850.

### 2.3. Statistical Analysis

An a priori sample size calculation was performed using G Power 3.1.9.7 based on a linear multiple regression model. Assuming a medium effect size (f^2^ = 0.20), a significance level of α = 0.05 and a statistical power of 0.80, with three predictors included in the model, the required total sample size was estimated to be 33 participants.

Descriptive statistics, including means and standard deviations or frequencies and percentages, were computed for all study variables. Skewness and kurtosis were examined to assess the normality of the distributions, and Cronbach’s α was calculated to evaluate the internal consistency of the scales. Associations among variables were analyzed using Pearson’s correlation coefficient. Subsequently, a series of hierarchical regression analyses were conducted to examine the impact of perfectionism in predicting ED-specific symptomatology (ERDC and GPMC) and general psychological symptoms (GSI). In the first model, age and BMI were entered in Step 1 as control variables, followed by the FMPS total score in Step 2. This analysis tested whether perfectionism contributed to explaining variance of EDRC independent of age and BMI. In the second model, GPMC was used as the dependent variable. Age and BMI were entered in Step 1, and the FMPS total score was added in Step 2. This model examined whether perfectionism contributed to general psychological maladjustment related to eating disorders after controlling for demographic factors. In the third model, GSI served as the dependent variable. Again, age and BMI were entered in Step 1, followed by FMPS total score in Step 2, to assess whether perfectionism contributed to explaining variance of overall psychological distress beyond age and BMI.

All analyses were performed using Jamovi (version 2.6.26).

## 3. Results

### 3.1. Descriptive Statistics of the Sample

Initially, 37 participants were recruited, but 2 were excluded due to incomplete questionnaires.

The final sample of the study included 35 Italian females diagnosed with AN (mean age ± SD: 21.9 ± 4.18 years; mean body mass index: BMI ± SD: 14.5 ± 1.77 kg/m^2^).

Descriptive statistics of the sample, including means and standard deviations for all study variables, are presented in Table 1.

### 3.2. Correlations Between Perfectionism Assessed with the Frost Multidimensional Perfectionism Scale (FMPS), the Eating Disorder Risk Composite (EDRC) Subscale of the EDI 3, the General Psychological Maladjustment (GPMC) Subscale of the EDI 3, and the Global Severity Index (GSI) of the SCL-90

Pearson’s r coefficients showed that age was negatively correlated with EDRC (r = −0.416, *p* = 0.013), indicating that younger participants tended to report higher EDRC scores. Age was not significantly associated with BMI (r = 0.001, *p* = 0.996), GPMC (r = −0.287, *p* = 0.094), GSI (r = −0.284, *p* = 0.099), or FMPS (r = −0.134, *p* = 0.443).

BMI showed a significant positive correlation with EDRC (r = 0.358, *p* = 0.038) and with FMPS (r = 0.497, *p* = 0.003), suggesting that higher BMI was associated with higher levels of these variables. The association between BMI and GPMC was not statistically significant (r = 0.321, *p* = 0.064), as well as the relationship between BMI and GSI (r = 0.258, *p* = 0.141).

EDRC was strongly and positively correlated with GPMC (r = 0.702, *p* < 0.001), GSI (r = 0.691, *p* < 0.001), and FMPS (r = 0.555, *p* < 0.001). Finally, GPMC showed a strong positive correlation with GSI (r = 0.807, *p* <0.001) and FMPS (r = 0.795, *p* < 0.001).

Correlations are presented in Table 2.

### 3.3. Hierarchical Linear Regression Models with Perfectionism Assessed with the Frost Multidimensional Perfectionism Scale (FMPS), the Eating Disorder Risk Composite (EDRC) Subscale of the EDI 3, the General Psychological Maladjustment (GPMC) Subscale of the EDI 3, and the Global Severity Index (GSI) of the SCL-90

A hierarchical multiple regression analysis was conducted to examine whether perfectionism, measured with the FMPS, significantly explained the variance of EDRC scores after controlling for age and BMI.

Age and BMI were initially entered in Model 1. The model was statistically significant, F(2, 31) = 6.71, *p* = 0.004, explaining 30.2% of the variance in EDRC scores. Age had a statistically significant negative influence (*p* = 0.009), indicating that higher age was associated with lower EDRC scores. BMI showed statistical significance (*p* = 0.023).

In Model 2, the total MPS score was added. The overall model remained significant, F(3, 30) = 8.03, *p* < 0.001, and explained 44.5% of the variance in EDRC scores. The addition of perfectionism significantly improved the model, ΔR^2^ = 0.143, F(1, 30) = 7.76, *p* = 0.009.

In the final model, FMPS total score was a significant factor (*p* = 0.009), indicating that higher levels of perfectionism were associated with higher EDRC scores. Age remained a significant negative factor (*p* = 0.014), whereas BMI was no longer statistically significant (*p* = 0.384). The results are presented in Table 3.

A hierarchical multiple regression analysis was conducted to examine whether perfectionism, measured with the FMPS, was significantly associated with GPMC scores after controlling for age and BMI.

Age and BMI were initially entered in Model 1. The model was statistically significant, F(2, 31) = 3.48, *p* = 0.043, explaining 18.3% of the variance in GPMC scores. However, age did not emerge as a statistically significant factor, while BMI showed a trend toward statistical significance.

In Model 2, the total FMPS score was added. The overall model was highly significant, F(3, 30) = 20.11, *p* < 0.001, explaining 63.5% of the variance in GPMC scores. The addition of perfectionism significantly improved the model, ΔR^2^ = 0.484, F(1, 30) = 43.8, *p* < 0.001.

In the final model, FMPS total score emerged as a strong and significant positive factor of GPMC (*p* < 0.001), indicating that higher levels of perfectionism were associated with higher GPMC scores. Neither age (*p* = 0.104) nor BMI (*p* = 0.509) were statistically significant factors. The results are presented in Table 4.

Finally, a hierarchical multiple regression analysis was conducted to examine whether perfectionism, measured with the FMPS, was significantly associated with psychological distress as indexed by the GSI of the Symptom Checklist-90-Revised (SCL-90-R), after controlling for age and BMI.

Age and BMI were initially entered in Model 1. The model was not statistically significant, F(2, 31) = 2.61, *p* = 0.089, explaining 14.4% of the variance in GSI scores. Neither age (*p* = 0.103) nor BMI (*p* = 0.130) had a statistically significant impact. In Model 2, the total FMPS score was added. The overall model was statistically significant, F(3, 30) = 8.48, *p* < 0.001, explaining 45.9% of the variance in GSI scores. The inclusion of perfectionism significantly improved the model, ΔR^2^ = 0.315, F(1, 30) = 17.5, *p* < 0.001.

In the final model, FMPS total score emerged as a significant positive factor associated with GSI (*p* < 0.001), indicating that higher levels of perfectionism were associated with greater psychological distress. Neither age (*p* = 0.162) nor BMI were statistically significant factors (*p* = 0.672). The results are presented in Table 5.

## 4. Discussion

The present study aimed at investigating the association between perfectionism and both eating disorder-specific symptomatology and general psychological symptoms in a sample of young adult Italian women diagnosed with AN.

As expected, the main findings of the study showed that perfectionism played a predictive role in both ED-related symptoms (EDRC and GPMC) and general psychological symptoms (GSI) in young adult women with AN. More specifically, perfectionism emerged not only as a correlate but as a significant predictor of both disorder-specific symptoms and broader psychological distress. Notably, its predictive role remained significant even after controlling for key variables such as age and BMI, underscoring its potential as an independent risk factor.

The role of perfectionism as a significant predictor of ED-specific symptomatology is consistent with a robust body of literature identifying perfectionism as a key factor in the onset and maintenance of eating disorder symptoms, as well as in the development of more severe eating psychopathology [24,25,26,27]. This association has been widely documented in adult populations [28], as well as in children and adolescents [29]. Our findings further extend this evidence by confirming the presence and relevance of this association in young adults, thereby corroborating the transdiagnostic role of perfectionism in ED [2].

Even when considering the predictive role of perfectionism on the general psychological symptoms (GSI) of young adult women with AN, the results showed similar patterns. Specifically, perfectionism significantly predicted general psychological symptoms, as measured by GSI of SCL-90, suggesting that its impact extends beyond eating-related pathology and it is also related to a more pervasive psychological maladjustment. As previously mentioned, the GSI of SCL-90 represents the most comprehensive indicator of an individual’s overall level of psychological distress by integrating information about the presence of symptoms and their perceived intensity across multiple domains such as depression, anxiety, somatization, and interpersonal sensitivity. This result is consistent with previous literature showing that maladaptive perfectionism is strongly associated with a wide range of psychological difficulties, including anxiety, depression, and global distress [10,28].

Overall, the study’s findings have several clinical implications. Specifically, they underscore the importance of systematically assessing perfectionism in individuals with ED, as it was confirmed to be a key factor contributing to both disorder-specific symptomatology and broader psychological distress. In addition, the present results may provide further evidence for adopting ED treatment approaches that go beyond mere symptom reduction and explicitly target underlying maintenance mechanisms, such as perfectionism. In this regard, Cognitive Behavioural Therapy for ED (CBT-E) provides a relevant framework, as it directly addresses both the disorder-specific and broader psychological processes in ED, such as perfectionism [2]. Clinical trials of CBT-E have demonstrated significant improvements in eating disorder symptoms, with concurrent decreases in associated cognitive features such as perfectionism, suggesting that these processes are functionally linked [30].

Finally, several limitations of the study warrant consideration. First, the relatively small sample size (*N* = 35) and the sampling method (recruiting only young adult women from a single clinical centre) may have limited the sample’s representativeness, thereby restricting the generalizability of the results.

Additionally, the cross-sectional design of the study precludes any causal inferences regarding the observed associations. Thus, the findings should be interpreted with caution. Another limitation of the present study concerns the exclusive use of self-report measures, which may be subject to several biases. In particular, social desirability bias may have influenced the participants’ responses. In addition, self-reported data may be affected by participants’ current mood state at the time of assessment. Future research could address these limitations by adopting a multi-method assessment approach, including clinician-administered interviews and behavioural or ecological momentary assessment methods.

A further limitation of the study concerns the potential overlap that exists between the construct of perfectionism measured via the FMPS and the perfectionism component included in the GPMC of the EDI 3. This overlap may introduce shared variance between measures, potentially inflating the strength of the observed associations. However, it is important to mention that the two instruments capture partially distinct aspects of perfectionism. The FMPS conceptualizes perfectionism as a multidimensional personality trait, encompassing both adaptive and maladaptive dimensions. In contrast, the perfectionism component included in the GPMC primarily reflects maladaptive aspects of perfectionism within a broader index of general psychological maladjustment, particularly in the context of eating disorder-related psychopathology.

Future replication of the study should aim to address these limitations by using larger samples to enhance the generalizability of the findings. In addition, the use of longitudinal or experimental designs would allow for the examination of causal relationships among the study variables. Future research should also consider employing multi-method assessment approaches, including behavioural or clinician-rated measures alongside self-report questionnaires. Finally, adopting a more representative sample would improve the external validity of the results.

## Figures and Tables

**Table 1:** Descriptive statistics of the study population.

	N	Means	SD	Min	Max
Age	35	21.9	4.18	15	33
BMI	35	14.5	1.77	10.0	17.8
EDRC	35	54.1	17.72	18	78
GPMC	35	133.6	52.39	15	224
GSI	35	1.89	0.759	0.078	3.29
FMPS	35	96.9	24.70	34	142

Note: BMI: body mass index; EDRC: Eating Disorder Risk Composite; GPMC: General Psychological Maladjustment Composite; FMPS: Frost Multidimensional Perfectionism Scale.

**Table 2:** Pearson’s correlation coefficients among the study variables.

	Age	BMI	EDRC	GPMC	GSI	FMPS
Age	—					
	—					
BMI	0.001	—				
	(*p* = 0.996)	—				
EDRC	−0.416 *	0.358 *	—			
	(*p* = 0.013)	(*p* = 0.038)	—			
GPMC	−0.287	0.321	0.702 ***	—		
	(*p* = 0.094)	(*p* = 0.064)	(*p* < 0.001)	—		
GSI	−0.284 (*p* = 0.099)	0.258 (*p* = 0.141)	0.691 *** (*p* < 0.001)	0.807 *** (*p* < 0.001)	— —	
FMPS	−0.134	0.497 **	0.555 ***	0.795 ***		—
	(*p* = 0.443)	(*p* = 0.003)	(*p* < 0.001)	(*p* ≤ 0.001)		—

Note: BMI: body mass index; EDRC: Eating Disorder Risk Composite; GPMC: General Psychological Maladjustment Composite; FMPS: Frost Multidimensional Perfectionism Scale. *: *p* < 0.05; **: *p* < 0.01; ***: *p* < 0.001.

**Table 3:** Hierarchical linear regression with EDRC as the dependent variable.

Predictors	B	SE	*p* Value	R^2^	Adjusted R^2^	ΔR^2^	F	*p* Value
Model 1				0.302	0.257		6.71	0.004
Age	−1.78	0.638	0.009					
BMI	3.64	1.524	0.023					
Model 2				0.445	0.143	0.143	8.03	<0.001
Age	−1.534	0.585	0.014					
BMI	1.410	1.596	0.384					
FMPS	0.318	0.114	0.009					

Note: BMI: body mass index; EDRC: Eating Disorder Risk Composite; GPMC: General Psychological Maladjustment Composite; FMPS: Multidimensional Perfectionism Scale.

**Table 4:** Hierarchical linear regression with GPMC as the dependent variable.

Predictors	B	SE	*p* Value	R^2^	Adjusted R^2^	ΔR^2^	F	*p* Value
Model 1				0.183	0.131		3.48	0.043
Age	−3.55	2.04	0.091					
BMI	9.63	4.86	0.057					
Model 2				0.668	0.635	0.484	20.11	<0.001
Age	−2.24	1.334	0.104					
BMI	−2.43	3.639	0.509					
FMPS	1,72	0.260	<0.001					

Note: BMI: body mass index; EDRC: Eating Disorder Risk Composite; GPMC: General Psychological Maladjustment Composite; FMPS: Frost Multidimensional Perfectionism Scale.

**Table 5:** Hierarchical linear regression with GSI as the dependent variable.

Predictors	B	SE	*p* Value	R^2^	Adjusted R^2^	ΔR^2^	F	*p* Value
Model 1				0.144	0.089		2.61	0.089
Age	−0.050	0.03	0.103					
BMI	0.111	0.07	0.130					
Model 2				0.459	0.404	0.315	8.48	<0.001
Age	−0.035	0.024	0.162					
BMI	−0.028	0.067	0.672					
FMPS	0.020	0.004	<0.001					

Note: BMI: body mass index; EDRC: Eating Disorder Risk Composite; GPMC: General Psychological Maladjustment Composite; FMPS: Frost Multidimensional Perfectionism Scale.

## Data Availability

Raw data will be uploaded to www.zenodo.org immediately after the manuscript is accepted, and they will be available upon reasonable request from the corresponding authors.
